# Supplementary material for: Neural synchronization is strongest to the spectral flux of slow music and depends on familiarity and beat salience
Source: eLife. 2022 Sep 12;11:e75515. doi: 10.7554/eLife.75515 (PMC9467512; doi:10.7554/eLife.75515)
Supplement: Transparent reporting form [file elife-75515-transrepform1.pdf]

1st Floor
24 Hills Road
Cambridge CB2 1JP, UK

P 01223 855340
W elifesciences.org
T @elife

eLife's transparent reporting form

We encourage authors to provide detailed information *within their submission* to facilitate the interpretation and replication of experiments. Authors can upload supporting documentation to indicate the use of appropriate reporting guidelines for health-related research (see EQUATOR Network), life science research (see the BioSharing Information Resource), or the ARRIVE guidelines for reporting work involving animal research. Where applicable, authors should refer to any relevant reporting standards documents in this form.

If you have any questions, please consult our Journal Policies and/or contact us: editorial@elifesciences.org.

Sample-size estimation

- You should state whether an appropriate sample size was computed when the study was being designed
- You should state the statistical method of sample size computation and any required assumptions
- If no explicit power analysis was used, you should describe how you decided what sample (replicate) size (number) to use

Please outline where this information can be found within the submission (e.g., sections or figure legends), or explain why this information doesn't apply to your submission:

This information can be found under Materials and Methods → *Participants*.

"Thirty-seven participants completed the study (26 female, 11 male, mean age = 25.7 years, SD = 4.33 years, age range = 19-36 years). Target sample size for this was estimated using G*Power3, assuming 80% power for a significant medium-sized effect. We estimate a target sample size of 24 (+ 4) for within-participant condition comparisons and 32 (+ 4) for correlations, and defaulted to the larger value since this experiment was designed to investigate both types of effects. The values in parentheses were padding to allow for discarding ~ 15% of the recorded data. The datasets of three participants were discarded because of large artefacts in the EEG signal (see section EEG data Preprocessing), technical problems and for not following the experimental instructions. The behavioral and neural data of the remaining 34 participants were included in the analysis."

Replicates

- You should report how often each experiment was performed
- You should include a definition of biological versus technical replication
- The data obtained should be provided and sufficient information should be provided to indicate the number of independent biological and/or technical replicates
- If you encountered any outliers, you should describe how these were handled
- Criteria for exclusion/inclusion of data should be clearly stated
- High-throughput sequence data should be uploaded before submission, with a private link for reviewers provided (these are available from both GEO and ArrayExpress)

Please outline where this information can be found within the submission (e.g., sections or figure legends), or explain why this information doesn't apply to your submission:

This information does not apply to our submission, as this study does not entail replicates.

eLife Sciences Publications, Ltd is a limited liability non-profit non-stock corporation incorporated in the State of Delaware, USA, with company number 5030732, and is registered in the UK with company number FC030576 and branch number BR015634 at the address 1st Floor, 24 Hills Road, Cambridge CB2 1JP | August 2014

Statistical reporting

- Statistical analysis methods should be described and justified
- Raw data should be presented in figures whenever informative to do so (typically when N per group is less than 10)
- For each experiment, you should identify the statistical tests used, exact values of N, definitions of center, methods of multiple test correction, and dispersion and precision measures (e.g., mean, median, SD, SEM, confidence intervals; and, for the major substantive results, a measure of effect size (e.g., Pearson's r, Cohen's d)
- Report exact p-values wherever possible alongside the summary statistics and 95% confidence intervals. These should be reported for all key questions and not only when the p-value is less than 0.05.

Please outline where this information can be found within the submission (e.g., sections or figure legends), or explain why this information doesn't apply to your submission:

Please find the attached table.

(For large datasets, or papers with a very large number of statistical tests, you may upload a single table file with tests, Ns, etc., with reference to sections in the manuscript.)

Group allocation

- Indicate how samples were allocated into experimental groups (in the case of clinical studies, please specify allocation to treatment method); if randomization was used, please also state if restricted randomization was applied
- Indicate if masking was used during group allocation, data collection and/or data analysis

Please outline where this information can be found within the submission (e.g., sections or figure legends), or explain why this information doesn't apply to your submission:

This information can be found in the first paragraph of the results section and under Materials and Methods → *Stimuli*.

"To be able to use a large variety of musical stimuli on the group level, and to decrease any effects that may have arisen from individual stimuli occurring at certain tempi but not others, participants were divided into four subgroups that listened to different pools of stimuli (for more details please see *Materials and Methods*)."

"Each participant was assigned to one of four pseudo-randomly generated stimulus lists."

Additional data files ("source data")

- We encourage you to upload relevant additional data files, such as numerical data that are represented as a graph in a figure, or as a summary table
- Where provided, these should be in the most useful format, and they can be uploaded as "Source data" files linked to a main figure or table
- Include model definition files including the full list of parameters used
- Include code used for data analysis (e.g., R, MatLab)
- Avoid stating that data files are "available upon request"

Please indicate the figures or tables for which source data files have been provided:

The Source data of the main results presented in the Figures 1-5 (+ figure supplements) are provided in the Source data files Figure 1 – Source Data 1, Figure 2 – Source Data1-3, Figure 3 – Source Data1, Figure 4 – Source Data 1 and Figure 5 – Source Data1.

***eLife*'s transparent reporting form: Statistical reporting**

Variable of Comparison	Section(s)	Test	N	Definition of center	Precision/ dispersion measure	p-value correction	Effect size
**Mutual Information (MI; MI data vs. MI surrogate × Tempo × Subgroup)**	Results → *Musical Features*<br><br>Materials and Methods → *Audio Analysis*<br><br>Figure 1D-E	Three-way ANOVA (MI data vs. MI surrogate × Tempo × Subgroup)	13 x 4	mean for test, median in Figure	25th+75th percentile	FDR	η^2
**SRCorr or TRF correlations across stimulation tempi**	Results → *Neural entrainment was strongest in response to slow music*<br><br>Materials and Methods → *Statistical Analysis*<br><br>Figure 2B, 3A	Repeated-measure ANOVA for every musical feature	34	mean	SEM	Greenhouse–Geiser correction (if applicable after Mauchly's test)	η^2
**Tempo-specificity of SRCorr or TRF correlations across musical features**	Results → *Spectral flux drives strongest neural entrainment*<br><br>Materials and Methods → *Data Analysis → EEG – Reliable Component Analysis*<br><br>Figure 2B, 3A	Linear Regression (per participant) + slope comparison with repeated-measure ANOVA	34	-	-	FDR	-

**SRCorr, SRCoh or TRF correlations across musical features**	Results → *Spectral flux drives strongest neural entrainment*<br><br>Materials and Methods → *Statistical Analysis*<br><br>Figure 2C, H, 3B	Repeated-measure ANOVA<br>+ Tukey's Test	34	mean for test, median in Figure	25th + 75th percentile	FDR	η^2
**Comparison of SRCorr or SRCoh between subgroups**	Results → *Spectral flux drives strongest neural entrainment*	One-way ANOVA	8-9 per subgroup	mean	-	FDR	η^2
**SRCoh across stimulation tempi**	Results → *Neural entrainment was strongest in response to slow music*<br><br>Materials and Methods → *Statistical Analysis*<br><br>Figure 2D-G	Repeated-measure ANOVA for every musical feature	34	mean for test, median in Figure	25th + 75th percentile	Greenhouse–Geiser correction (if applicable after Mauchly's test)	η^2
**Comparison of SRCoh at stimulation tempo vs. first harmonic**	Results → *Neural entrainment was strongest in response to slow music*<br><br>Materials and Methods → *Statistical Analysis*<br><br>Figure 2I	Paired-sample t-test	34	mean for test, median in Figure	25th + 75th percentile	FDR	r_e

**Comparison of the FFT amplitude of the stimuli at stimulation tempo vs. first harmonic**	Results → *Neural entrainment was strongest in response to slow music*<br><br>Figure 2J	Paired-sample t-test	13	mean for test, median in Figure	25th + 75th percentile	FDR	r_e
**Significant time-lag window of TRF weights**	Results → *Spectral flux drives strongest neural entrainment*<br><br>Materials and Methods → *Data Analysis* → *EEG – Temporal Response Function*<br><br>Figure 3C-F; Figure 3 – figure supplement 1D-K	Cluster-based permutation testing	34	mean	-	-	-
**Latency analysis of the significant time window of the TRF weights**	Results → *Spectral flux drives strongest neural entrainment*<br><br>Materials and Methods → *Data Analysis* → *EEG – Temporal Response Function*<br><br>Figure 3 – figure supplement 1G, K	Linear regression	34	mean	95% confidence bounds	-	R^2
**Comparison of TRF correlations and SRCorr**	Results → *Results of TRF and*	Linear mixed-effects model for	34	-	-	FDR	R^2

**Or**<br><br>**Comparison of TRF correlations and SRCoh at the stimulation tempo or first harmonic**	*SRCorr/SRCoh converge*<br><br>Materials and Methods → *Data Analysis* → *EEG – Comparison of TRF and RCA measures*<br><br>Figure 4, Figure 4 – figure supplement 1	every musical feature					
**Stimulation tempo effects on behavioral ratings**	Results → *Familiar songs and songs with an easy-to-tap beat drive strongest neural entrainment*<br><br>Materials and Methods → *Data Analysis* → *Behavioral data*<br><br>Figure 5A	Repeated-measure ANOVA	34	mean	SEM	Greenhouse–Geiser correction (if applicable after Mauchly's test)	η^2
**Comparison of TRF correlations of low vs. high rated trials (enjoyment, familiarity and beat tapping difficulty)**	Results → *Familiar songs and songs with an easy-to-tap beat drive strongest neural entrainment*<br><br>Materials and Methods → *Data Analysis* → *EEG –*	Paired-sample t-test	34	mean for test, median in Figure	25th + 75th percentile	FDR	r_e

	Temporal Response Function<br><br>Figure 5C-F						
**Impact of musical training on TRF correlations**	Results → *Familiar songs and songs with an easy-to-tap beat drive strongest neural entrainment*<br><br>Materials and Methods → *Participants*<br><br>Figure 5 – figure supplement 2	Pearson correlation	34	mean	-	-	R
**Tapping Behavior**	Results → *Brain responses to musical features predict perceived beat rate*<br><br>Figure 5 – figure supplement 3	Fitted Skewed Gaussian	29	mode	lambda	-	-
